# Antisense oligonucleotide suppression of human IGF-1R inhibits the growth and survival of in vitro cultured epithelial ovarian cancer cells

**DOI:** 10.1186/1757-2215-6-71

**Published:** 2013-10-08

**Authors:** Jie Tang, Junjun Li, Guqing Zeng, Yanxiang Tang, Wenfang Tian, Jie He, J Philippe York, Xuefeng Xia

**Affiliations:** 1 Department of Gynecologic Oncology, Hunan Provincial Tumor Hospital, Xianjiahu Rd, Changsha, Hunan 410006, China; 2 Department of Pathology, Hunan Provincial Tumor Hospital, Changsha, China; 3 Department of General Surgery, School of Medicine, University of South China, Hengyang, China; 4 Houston Methodist Research Institute, Weill Cornell School of Medicine, 6670 Bertner Ave, Houston, TX 77030, USA; 5 The Third Affiliated Hospital, Guangzhou Medical University, Guangzhou, Guangdong 510150, China

**Keywords:** Antisense, IGF-1R, Epithelial ovarian cancer

## Abstract

**Background:**

Preclinical evaluation of the anti-neoplastic activity of antisense oligonucleotide (AS) suppression of human insulin-like growth factor I receptor (IGF-IR) in human epithelial ovarian cancer (EOC).

**Methods:**

Ovarian cancer cells from 36 patients with EOC were investigated under serum-free tissue culture conditions. IGF-I production was evaluated by standard ELISA. IGF-IR and phosphorylated IRS-1, AKT, and MAP kinase expression and protein levels were evaluated by immunohistochemistry and Western blotting. Cancer cell growth and proliferation assays were performed in triplicates using MTT assay. Apoptosis was evaluated by TUNNEL assay.

**Results:**

All ovarian cancer tissue samples tested produced IGF-I and expressed IGF-IR, supporting the existence of an autocrine loop. Treatment of primary ovarian cancer cell lines with an IGF-1R AS inhibited growth and proliferation and decreased clonogenicity in soft agar assay. AS treatment was demonstrated to inhibit the expression of IGF-1R and decrease the concentration of phosphorylated IRS-1, AKT, and MAP kinase signaling protein downstream of the IGF-IR. We also observed that the IGF-1R AS sensitized cancer cell lines to cisplatin *in vitro* through the PI3K pathway.

**Conclusions:**

IGF-IR enhances the proliferation and tumorigenicity of human ovarian cancer cells and inhibition of IGF-IR by AS oligonucleotide treatment potentiates the activity of cisplatin *in vitro*. Therefore, IGF-1R is a potential molecular target in ovarian cancer.

## Background

Epithelial ovarian cancer (EOC) constitutes 90% of ovarian malignancies and is the most lethal gynecological malignancy. Although most EOC patients experience a reasonable initial clinical response to debulking surgery and chemotherapy, the majority of these patients will not be cured [1-3]. Approximately 70% of EOC patients will experience EOC recurrence and chemoresistance is responsible for the majority of ovarian cancer-related deaths [1,4,5]. Current treatments are incapable of curing recurrent ovarian carcinomas due to their rapid evolution into chemoresistant disease. Therefore, new therapeutic modalities are urgently needed to overcome chemoresistance in EOC. Accumulating evidence suggests that the insulin like growth factor (IGF) pathway may be a good therapeutic target in several cancer types, including ovarian cancer [5-7]. In this paper, we will focus on the role of IGF-1R in ovarian cancer tumorigenesis and treatment.

IGF ligands, receptors and IGF binding proteins (IGFBPs) have been shown to play a critical role in the development and progression of human cancers [5,8,9]. IGFBPs are a family of six homologous proteins with high binding affinity for IGF-I and IGF-II. All six IGFBPs have been shown to inhibit IGF action, but stimulatory effects have also been established for IGFBP-1, -3, and -5 [10]. Elevated plasma concentrations of IGF-1 or IGFBP-3 have been associated with several types of cancers, including breast, prostate and lung cancer [11-13]. In addition, IGF-1/IGF-IR has been studied extensively in metastatic colon, pancreatic, prostate and breast cancer [14,15]. In many human cancers, there is a strong association between dysregulation of the IGF signaling pathway and cancer risk that has been extensively investigated [7,15-17]. In contrast to other epithelial malignancies, limited data are available on the potential role of the IGF-1R in EOC [18].

Yee et al. identified expression of IGF-I mRNA in 3 of 10 ovarian cancer cell lines (OVCAR-3, OVCAR-7 and PEO4) and ovarian cancer tissue specimens [5]. They also reported expression of several IGFBPs and IGF-IR by ovarian cancer cells. This study suggested that all necessary components for an IGF-I-mediated autocrine loop are present in ovarian cancer cells, an observation that was confirmed in an early study using the OVCAR-3 cell line [17]. During the same period, it was reported that IGF-I level was higher in cyst fluid from invasive malignant neoplasms compared to benign tumors [17]. Later, another group confirmed the presence of IGF-IR expression by immunohistochemistry (IHC) in 100% of the ovarian carcinoma samples tested [6]. These initial studies opened the door to further research in ovarian cancer, indicating an involvement of the IGF system in ovarian tumorigenesis [11,14,15,19]. Moreover, Brokaw et al. showed that high free IGF-I protein expression in ovarian tumor tissue was independently associated with the progression of ovarian cancer [19]. IGF-I mRNA expression was also associated with disease progression, implying that both endocrine and paracrine/autocrine regulations of IGF-I activity are involved in ovarian cancer [6,20]. Similarly, microarray expression profiles from 64 EOC patients demonstrated that individual genes, including IGF-I, IGF-IR and several genes downstream of the receptor, were over-expressed in tumors associated with an unfavorable prognosis [21].

The strategies to target IGF-1 system in cancer consist of [1] reducing circulating ligand levels or bioactivity [2,22] blocking receptor function using receptor-specific antibodies or small-molecule tyrosine kinase inhibitors [1,23,24] and [3] activating AMP-activated protein kinase (AMPK) [25]. In the past few years, various inhibitors of IGF-IR have been developed, including AMPK activators [26]. *In vivo* studies have expanded the understanding of the IGF-1 system and its therapeutic potential in cancer; however, *in vitro* results have been inconclusive and further study is needed to more precisely determine the therapeutic significance of these findings.

In this study, through an in vitro culture system as an experimental model we used tumor specimens and primary ovarian cancer cells from patients with advanced-stage epithelial ovarian cancer to comprehensively analyze the possibility that IGF-1R is important in regulating the autocrine growth of ovarian cancer cells. We report that the ovarian cancer cells from patients with advanced EOC produce endogenous IGF-1, express IGF-1R and grow autonomously in serum-free media (SFM). Their growth in these conditions, however, is further stimulated by the addition of IGF-1. Treatment with IGF-1R mRNA antisense oligodeoxynucleotides (AS) markedly inhibits the proliferation of these cells in SFM in the presence of IGF-1. This inhibition corresponds to a reduction in the amount of detectable IGF-1R. These data together suggest that the IGF-1/IGF-1R system may have a prominent role in the proliferation of EOC cells. The results also support another function of the IGF-1R to protect cancer cells from apoptosis. We also observed that IGF-1R AS sensitized cells to cisplatin *in vitro.*

## Methods

### Patient and tumor samples

The study population consisted of 36 patients with advanced EOC (International Federation of Gynecology and Obstetrics, FIGO stage III/IV) from Hunan Provincial Tumor Hospital, diagnosed between 2007 and 2008. The characteristics of the patient cohort are described in Table 1. All patients underwent total abdominal hysterectomy and bilateral salpingo-oophorectomy by an expert gynecologic oncologist according to standard debulking guidelines and all received standard adjuvant platinum/taxane-based chemotherapy. Outpatient follow-up was regularly performed (median follow-up duration was 3 years). Tissue and clinical data collection was approved by the Institutional Review Boards in Hunan Provincial Tumor Hospital, and all patients provided informed consent. Ovarian cancer samples were collected at the time of primary debulking surgery and frozen at −80°C. Staging was reported according to the FIGO (2009). Optimal debulking was defined as ≤1 cm gross residual disease, and suboptimal debulking as more than 1 cm residual disease. Overall survival (OS) was defined as the time between the date of diagnosis of ovarian cancer and the date of death.

**Table 1:** **Clinical/pathological characteristics of the patients**^**a **^**(*n *=36)**

**Characteristic**	**Number (%)**
Age (median, range)	57(37–72)
Stage (FIGO)	
III	31(85%)
IVA	5(15%)
Debulking Status	
Optimal (≤1 cm)	24(66%)
Suboptimal (>1 cm)	12(34%)
Histological type	
Serous	32(90%)
Endometriod	2(5%)
Clear cell	2(5%)
Grade	
2	7(20%)
3	29(80%)

^a^All patients received standard adjuvant platinum/taxane-based chemotherapy.

### Preparation of patient tumor cell lines

Tumor tissue from each patient was cut into small pieces with a sterile scalpel blade. Tumor fragments were suspended in 2 mL of a 0.25% Trypsin-EDTA solution (Gibco-BRL, Grand Island, NY) and pipetted up and down to break up large tissue fragments. After incubation for 5 minutes at 37°C, the suspension was again disaggregated and transferred to a 100 mm Primaria Petri dish (BD, Franklin Lakes, NJ) containing 10 mL of RPMI 1640/Opti-MEM (Gibco-BRL) (1:1 ratio) supplemented with 10% fetal bovine serum, 2 mmol/L glutamine, 100 units/mL of penicillin, and 100 μg/mL streptomycin. Cell lines were passaged three to four times before use. Normal epithelial ovarian cells (NEOC) from 10 patients with benign serous epithelial ovarian tumor were tested as a normal control. Tumor cells (10^9^) were harvested, rinsed, re-suspended in 2 mL HBSS and lysed by five freeze/thaw cycles (dry ice/room temperature), followed by sonication for 2 minutes at 4°C to break up DNA. Debris was spun down at 400 × g for 25 minutes, and the supernatant was collected and stored in aliquots at −80°C. Lysates from normal epithelial ovarian cells were also used as a normal control.

### Immunohistochemistry(IHC) staining of EOC tissue samples

Immunohistochemistry(IHC) was performed to determine protein expression of IGF-1R in EOC surgical specimens, using monoclonal antibodies against IGF-1R (mouse monoclonal, 1:100; NeoMarkers, Fremont, CA, USA). The slides were then incubated with a goat biotinylated secondary antibody provided in the detection kit, and horseradish peroxidase agent for 60 min, and incubated with the substrate chromogen 3,3′-diaminobenzidine for 5 min. Protein expression levels were assessed by two experienced pathologists.

### Level of total IGF-I in tumor cell lysates and cell culture medium

A non-extraction IGF-1 enzyme-linked immunosorbent assay (ELISA) kit (DSL-10-2800) from Diagnostics Systems Laboratories, Inc., Webster, TX, USA was used for the determination of IGF-1 level in tumor cell lysate and cell culture medium. Samples were tested in duplicates and repeated if the correlation coefficient between the absorbance and the amount in the standards was less than 0.95.

### Cell growth and proliferation analyses

Cancer cells and normal epithelial ovarian cells were seeded in medium containing 10% fetal bovine serum, at an initial density of 3–6 × 10^3^ cells/cm^2^. The cells were allowed to attach for 24 h and then arrested in serum free medium (SFM). After the addition of 20 ng/ml IGF-1 (Life Technologies, Inc., Gaithersburg, MD), duplicate cultures were counted in a hemocytometer at 24, 48 and 72 h. In a successive experiment, normal cells and cancer cells were tested for growth in SFM without the addition of IGF-1. Then, cancer cells maintained in SFM were exposed to an IGF-1R sense oligonucleotide (5′-AAG TCT GGC TCC GGA GGA) or antisense (5′-TCC TCC GGA GCC AGA CTT) IGF-1R mRNA oligodeoxynucleotides (40 ng/ml) for 48 h with and without the addition of IGF-1. This antisense represents codons 2-7of the prepropeptide and has been shown previously to effectively decrease the number of IGF-1 receptors [27]. In this experiment, the cells were counted using MTT (Sigma Chemical Co., Milwaukee, WI). This assay is based on reduction of MTT to formazan by enzymes present only in viable, metabolically active cells. After exposure to sense or antisense IGF-1R mRNA oligonucleotides, the medium was removed, and 0.1 ml of MTT (50 μg) was added to each well. After incubation for 2 h at 37°C, the multiwell plate was centrifuged to pellet formazan crystals, and the medium was discarded. Then, 0.1 ml of DMSO was added to each well to dissolve the MTT formazan crystals. The absorbance of formazan at 540 nm was measured by Emax microplate reader. A mean of 18 replicates cultured from three experiments were used for statistical analysis.

### Western blot analyses

The expression of IGF-1R and phosphorylated IRS-1, AKT and MAP kinase in cancer cells was analyzed by Western immunoblotting as follows: cell lysates were obtained from the above cells exponentially growing in 10% serum. After clarification by centrifugation and protein concentration determination, 20 μg of protein were resolved on a 4–15% polyacrylamide gradient gel by SDS-PAGE and electroblotted into a nitrocellulose filter. The filter was immunoblotted with mouse anti-human monoclonal antibodies to identify the IGF-1R α and phosphorylated IRS-1, AKT and MAP kinase (Life Technologies, Inc.), followed by secondary horseradish peroxidase- conjugated horse anti-mouse IgG antibodies (Oncogene, Science, Inc. Mineola, NY). Antigen bound to nitrocellulose membrane polyvinylidene difluoride (PVDF) was detected and protein bands were visualized by enhanced chemiluminescence with a diaminobenzidine tetrahydrochloride (DAB) substrate.

### Apoptosis assays

Apoptosis was determined by TUNEL, using an in-situ cell-death detection kit (Boehringer Mannheim, Indianapolis, IN) according to the manufacturer's protocol. Briefly, cancer cells were treated with 40 ng/ml antisense IGF-1R mRNA (AS), LY294002, cisplatin or the combination of these two agents for 48 to 72 hours. After treatment, cells were trypsinized and cytospin preparations were obtained. Cells were fixed with freshly prepared paraformaldehyde [4% in PBS (pH 7.4)], rinsed with PBS, and incubated in permeabilization solution. After cross-reaction with TUNEL reaction mixture for 60 min at 37°C and cross-reaction with converter-alkaline phosphatase solution for 30 min at 37°C in a humidified chamber, the slides were reacted with alkaline phosphatase substrate solution for 5–10 min (Vector Laboratories, Burlington, MA), rinsed and mounted under a coverslip for analysis with a light microscope. The number of TUNEL-positive cells was counted in five different fields under a light microscope at ×40 magnification, and representative fields were photographed. The percentages of apoptotic cells were calculated from the ratio of apoptotic cells to total cells counted. At minimum, 500 cells were counted in five different fields, and assays were performed in duplicate three times (×6).

### Clonogenicity in soft agar

After exposure to antisense IGF-1R mRNA oligonucleotides (40 ng/ml) for 48 h, cancer cells were seeded at a density of 6 × 10^3^/35-mm plate in 15% fetal bovine serum on a top layer of 0.3% agar and a bottom support layer of 1% agar. The plates were incubated at 37°C. Colonies of greater than 10 cells were counted 7 and 14 days later. Cancer cells without antisense treatment were used as a control.

### Statistical analyses

Comparisons between case subjects and control subjects were undertaken by t tests, χ^2^ tests and 2 independent samples tests. Differences were considered significant when P < 0.05.

## Results

### Clinical and pathological characteristics

The clinical and pathological characteristics of the 36 patients are shown in Table 1. The median patient age was 57 years. All patients had advanced-stage disease, and the majority had grade 3 (80%) or serous (90%) EOC. After primary surgery, 66% of patients were optimally debulked. The median follow-up interval was 3 years. As of January 2011, 20 patients from the entire cohort remained alive.

### IGF-1R and IGF-1 expression in EOC cells

**Figure 1:**
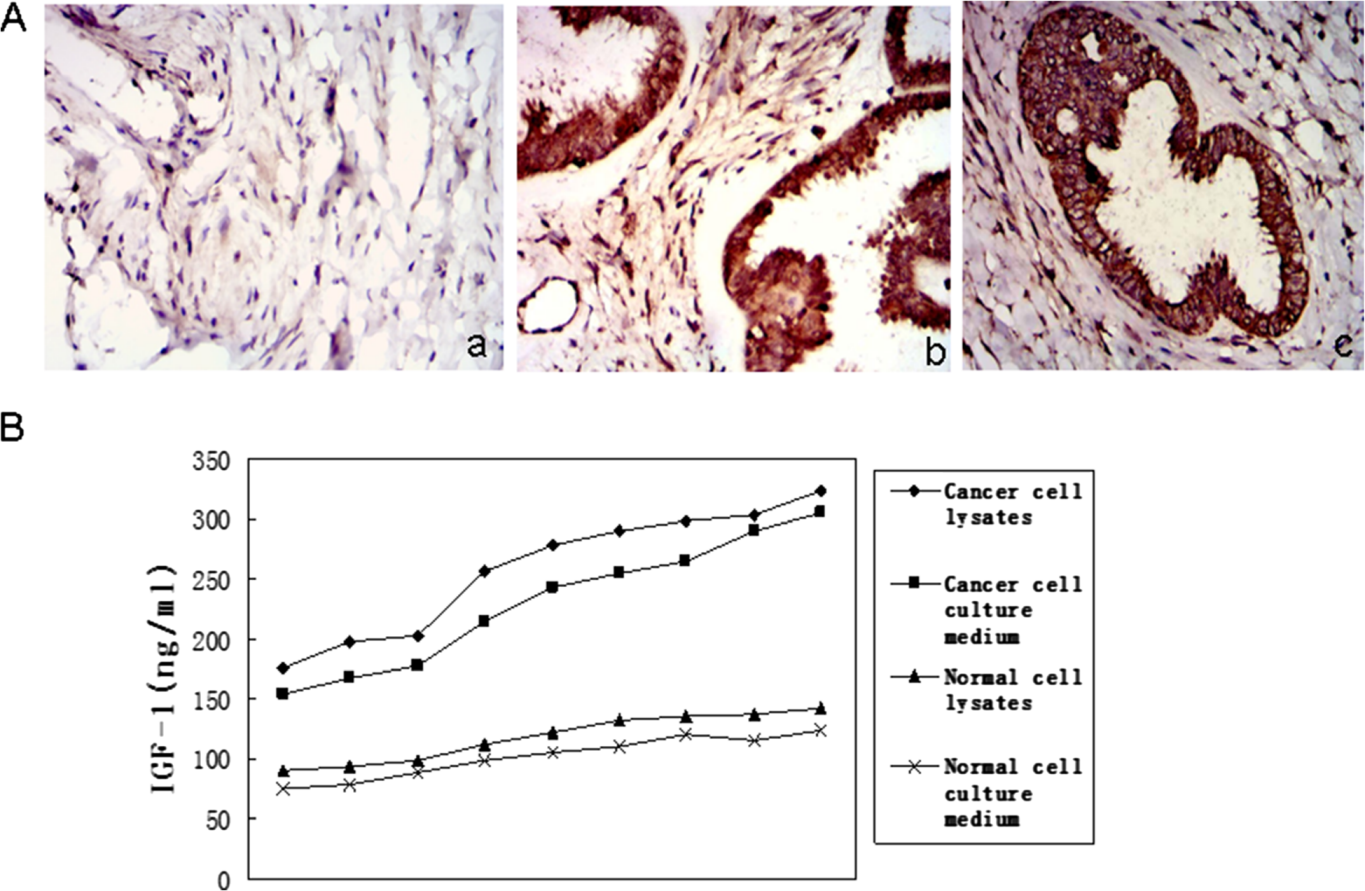
**Level of IGF-1R and IGF-1 in EOC. (A)** IGF-1R expression in surgical specimens of epithelial ovarian cancer (EOC). Representative pictures of surgical samples with different IGF-1R expression (original magnification ×100). **(a)** IGF-1R protein expression was low in benign epithelial serous ovarian tumor tissues. **(b,c)** EOC tissues clearly expressed IGF-1R protein. Magnification was in small square. **(B)** Quantitated IGF-1 level in tumor cell lysates and cell culture medium by ELISA. IGF-1 level in tumor cell lysates and cell culture medium is much higher than in normal epithelial ovarian cell lysates and cell culture medium(n=10)(P<0.05). [2 independent sample tests].

We examined the expression of IGF-1R in surgical specimens of epithelial ovarian cancer by IHC. As shown in Figure 1A-a, IGF-1R protein staining was sparse in both normal and benign serous epithelial ovarian tumor tissues in all surgical specimens examined (n=10). In contrast, IGF-1R staining was significantly increased in epithelial ovarian cancer tissues (n=36) (Figure 1A-b, c). All specimens were graded for the distribution of IGF-1R staining. Low grade indicates that the distribution of IGF-1R staining is less than 50% of tumor area, whereas high grade indicates that the distribution of IGF-1R staining is more than 50% of tumor area. 25 of 36 epithelial ovarian cancer tissues expressed IGF-1R with high grade, and 9 expressed IGF-1R with low grade. After testing 10 samples from each group using ELISA, we observed that primary cancer cell lines had consistently higher levels of IGF-1 in both cell lysates and cell culture medium than normal epithelial ovarian cell lines (P<0.05) (Figure 1B). The mean level of IGF-1 in cell lysates and cell culture medium was higher among case subjects (258.2 ng/mL/230 ng/ml) than among control subjects (118.3 ng/mL/101.9 ng/ml) (P <0.05).

### Growth characteristics of ovarian cancer cells

**Figure 2:**
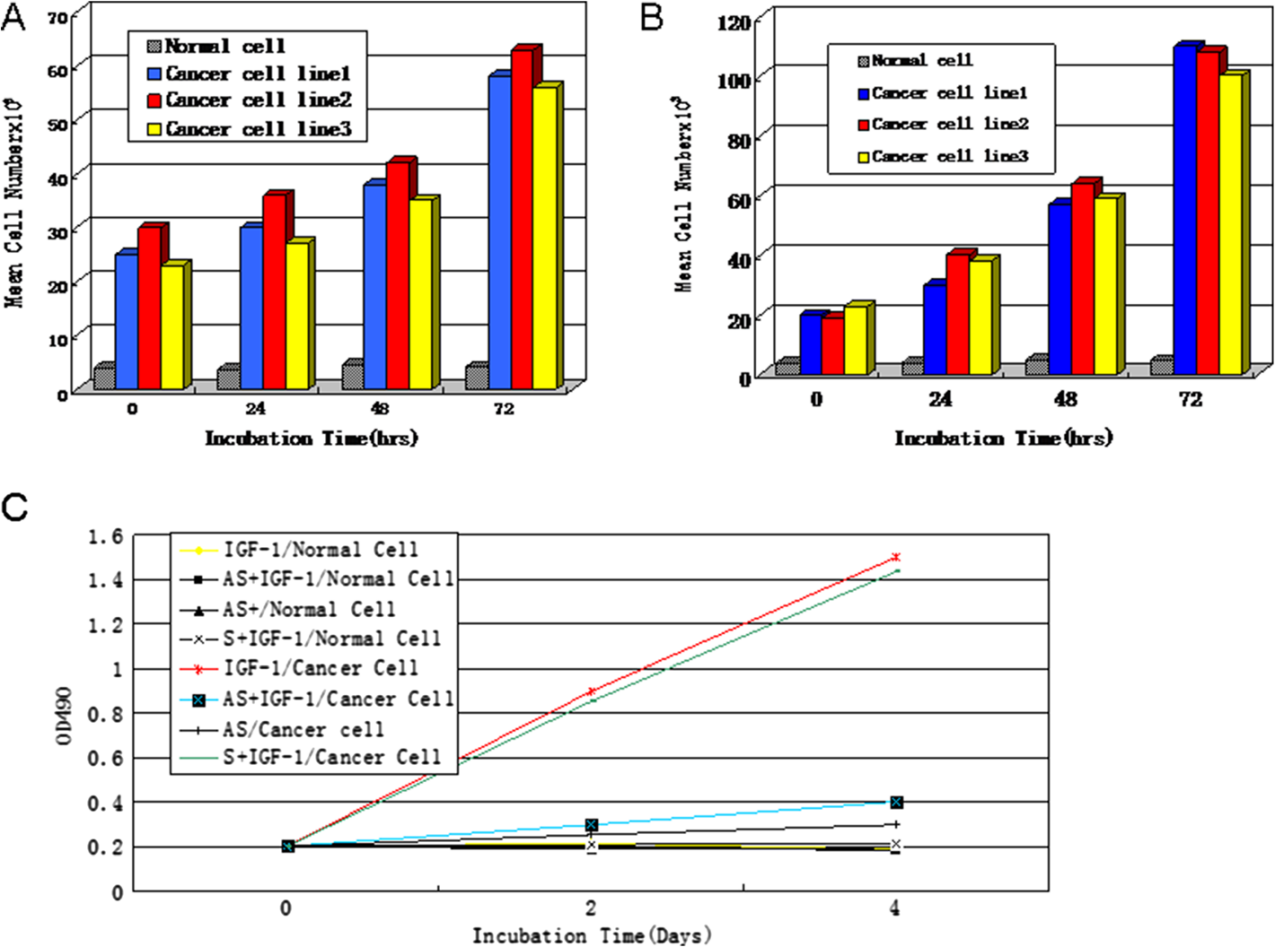
**Proliferation characteristics of ovarian cancer cells.** Proliferation of normal cells and cancer cell lines 1, 2, and 3 in serum free medium (SFM) with **(A)** and without **(B)** the addition of IGF-1 (20 ng/ml). The proliferation of cancer cell lines is approximately 20-fold over that of normal cells (P<0.05). **(C)** Effect of antisense (AS) IGF-1R mRNA oligonucleotides on the growth of cancer cells. Cancer cells were maintained in SFM as described in the “Methods” section. AS or sense oligonucleotides (S,40 ng/ml) were added to the medium for 24 h before the addition of IGF-1 (20 ng/ml) at time zero. Cells were counted at the indicated times. The data represent the mean of triplicate determinations from one of three representative experiments.

The growth characteristics of cancer cells compared with normal cells are shown in Figure 2A. When cultured in SFM, normal cells did not respond to the addition of exogenous IGF-1. Conversely, the growth response of cancer cells to IGF-1 was 20-fold greater (P < 0.05). Even in SFM alone, cancer cells proliferated, although at a lower rate (8-fold, Figure 2B) (P < 0.05). The addition of AS against the IGF-1R mRNA decreased cancer cell growth rate by 70%, both in the presence and absence of exogenous IGF-1 (Figure 2C) (P < 0.05). We observed IGF-IR AS growth inhibition of the ovarian cancer cells, with IC50 between 25 and 30 ng/ml.

### IGF-1R and phosphorylated AKT expression on cancer cells after IGF-IR AS treatment

**Figure 3:**
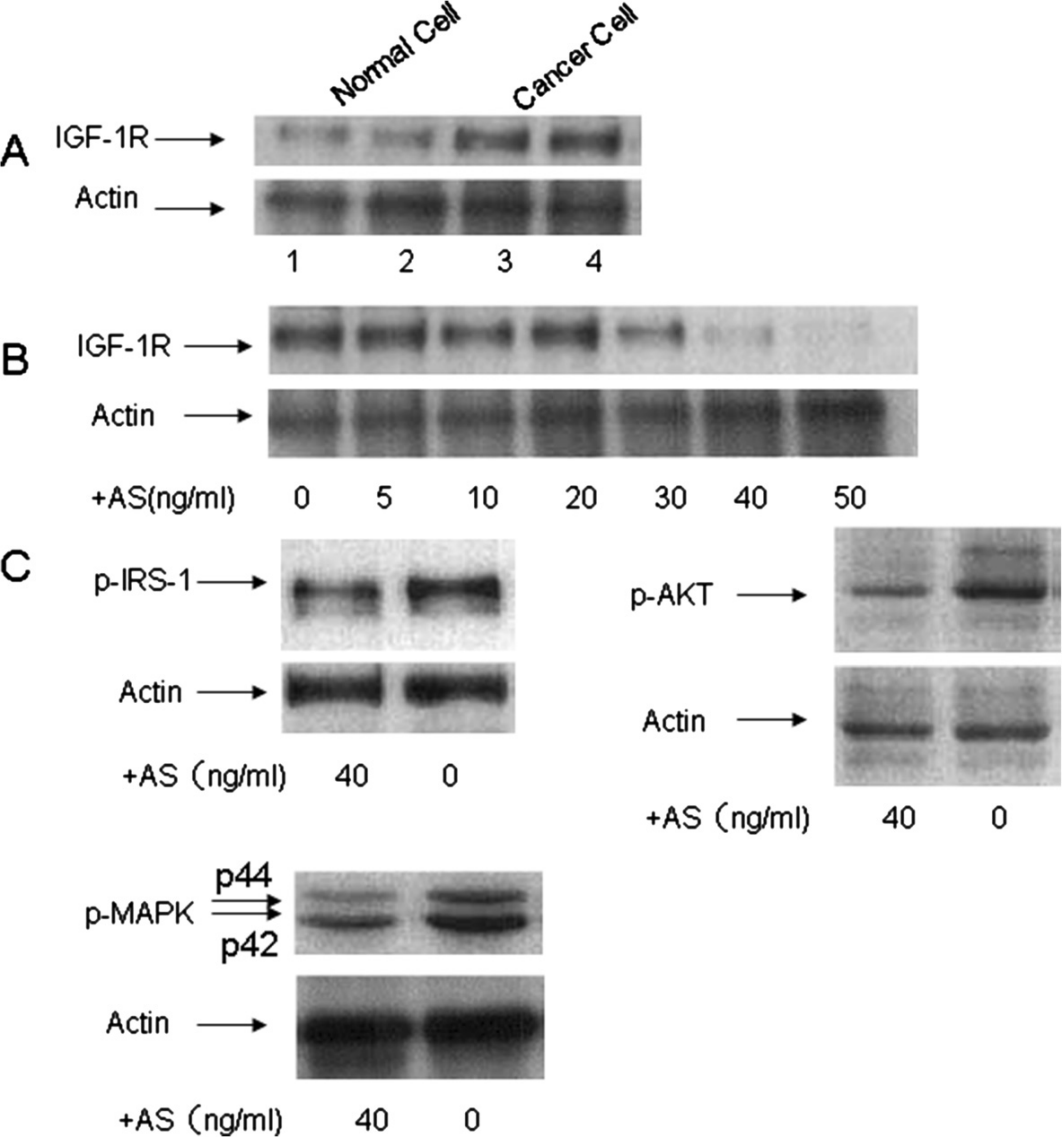
**Effect of antisense IGF-1R mRNA oligonucleotides (AS) on IGF-1R expression by cancer cells.** Cancer cells were arrested in serum free medium (SFM) as described in the “Methods” section for 24 hrs. Oligonucleotides at different doses were added to the medium for 48 hrs before cells were harvested for western blotting assay. **(A)** Western Blotting showed the cancer cells expressing more IGF-1R than the normal epithelial ovarian cells. 1 and 2 represent normal epithelial ovarian cells; 3 and 4 represent epithelial ovarian cancer cells. Actin was used for the loading control. **(B)** AS inhibited IGF1-R expression at different concentrations in epithelial ovarian cancer cells. Actin was used for the loading control. **(C)** AS also variably inhibited phosphorylated IRS-1, AKT, and MAP kinase expression in epithelial ovarian cancer cells. Actin was used for the loading control.

Ovarian cancer cells exhibited a distinct band of molecular weight 135,000 representative of the α subunit of the IGF-1R when examined by Western immunoblotting. Conversely, at the same molecular weight normal epithelial ovarian cells displayed a weak, indistinct band (Figure 3A). After incubating cancer cells with IGF-IR AS for 48 hrs in SFM, IGF-1R expression was effectively inhibited by at 30-50 ng/ml IGF-1R mRNA oligonucleotides (Figure 3B). We observed expression inhibition of IGF-1R by IGF-IR AS, with IC50 of 30 ng/ml. Interestingly, the IGF-IR AS also variably decreased the expression of the phosphorylated IRS-1, AKT, and MAP kinase signaling proteins downstream of IGF-IR (Figure 3C).

### Sensitization of ovarian cancer cells to cisplatin by IGF-IR AS

**Figure 4:**
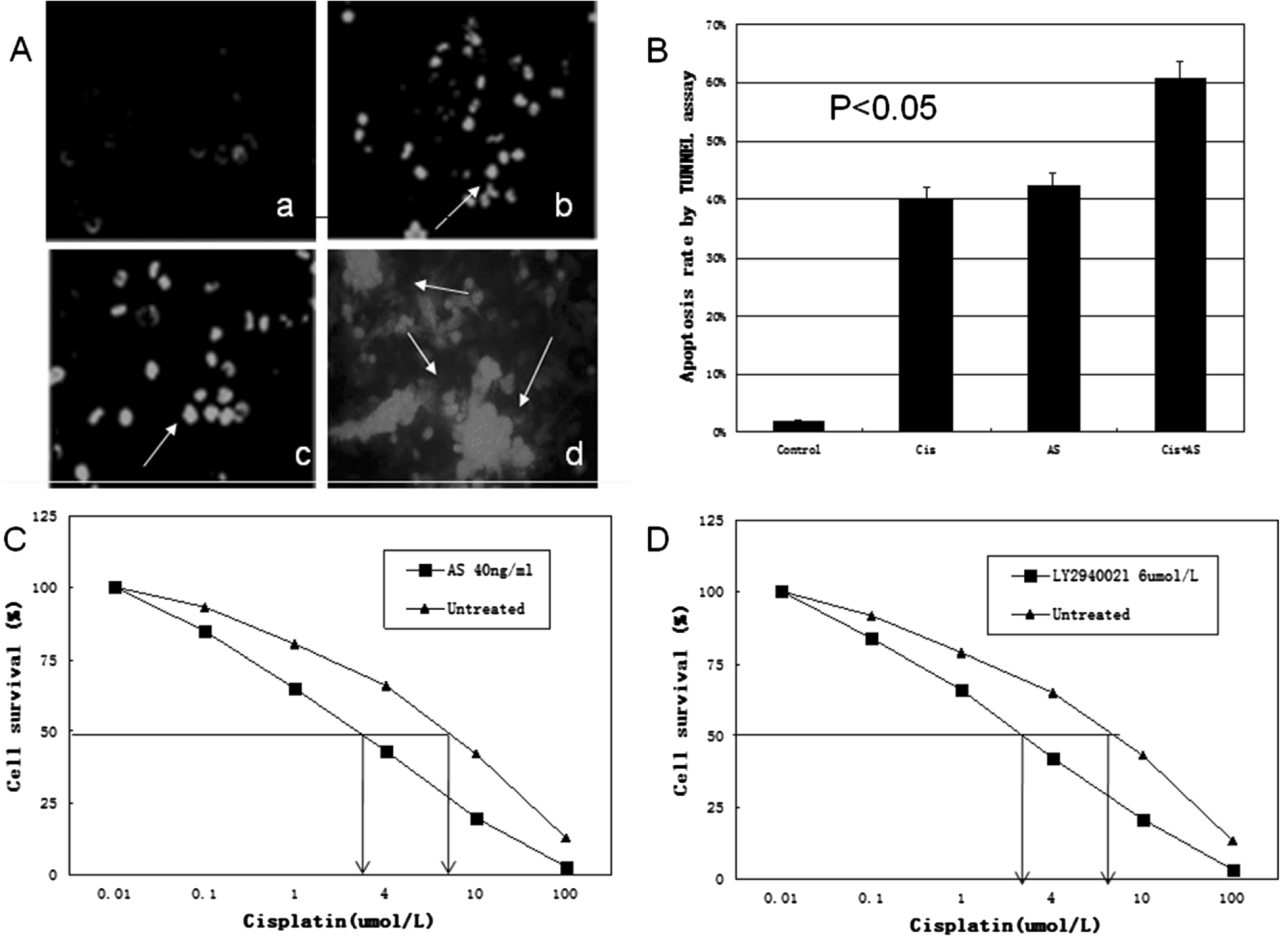
**Apoptosis in human ovarian cancer cells was induced by different treatments as revealed by TUNEL assay. (A)** Apoptosis was shown in human oravian cancer cells by TUNEL assay. TUNEL-positive nuclei due to DNA fragmentation (white condensed spots) are indicated by arrows. **(a)** Without treatment as a negative control; **(b)** Treated with cisplatin (Cis) alone; **(c)** Treated with antisense IGF-1R mRNA oligonucleotide (AS) alone; (d) Treated with the combination (Cis+AS). **(B)** There was a significant potentiation in the induction of apoptosis observed in cancer cells treated with both Cis and AS as compared to cells treated with either agent alone (P<0.05). **(C)** Effect of IGF-IR (AS) on cisplatin treated ovarian cancer cells. Ovarian cancer cells were cultivated in the presence of AS and cisplatin. Arrows, IC50 concentrations of cisplatin for AS (left arrow) and medium control (right arrow). **(D)** IGF-IR signaling through the PI3K pathway is essential for cisplatin resistance in ovarian cancer cells. Ovarian cancer cells were cultivated in the presence of cisplatin and LY294002, which inhibits PI3K activity. Arrows, IC50 concentrations of cisplatin for LY294002 and medium control.

A TUNEL alkaline phosphatase assay demonstrated that ovarian cancer cells underwent apoptosis after exposure to cisplatin, a first line chemotherapy agent for EOC (Figure 4A-b). To test whether the observed IGF-1R activation plays a role in cisplatin-induced apoptosis, we selectively inhibited IGF-1R activity by using IGF-IR AS. Apoptosis was also induced in ovarian cancer cells after IGF-IR AS treatment (Figure 4A-c). However, 24 hours pretreatment with 40 ng/ml AS followed by the treatment of 2umol/L cisplatin for 48 hours significantly increased apoptosis compared to either agent alone (Figure 4A-d and 4B). These results thus indicate that the IGF-1R is required for human EOC cells undergoing apoptosis upon cisplatin treatment.

### IGF-IR signaling through the PI3K pathway is crucial for cisplatin resistance

To determine the function of IGF-IR and PI3K signaling pathways in cisplatin resistance, we used specific inhibitors. As shown in Figure 4C, the PI3K inhibitor LY294002 [28] efficiently sensitized ovarian cancer cells to cisplatin, similar to IGF-1R AS treatment (Figure 4D). In contrast, inhibition of ERK1/2 signaling by the MAP/ERK kinase 1/2 inhibitors U0126 [29] did not affect cisplatin resistance (data not shown), indicating that the ERK1/2 signaling pathway is not involved. These results indicate that IGF-IR dependent signaling through the PI3K pathway is responsible for cisplatin resistance.

### Effect of IGF-1R AS on clonogenicity of cancer cells

**Figure 5:**
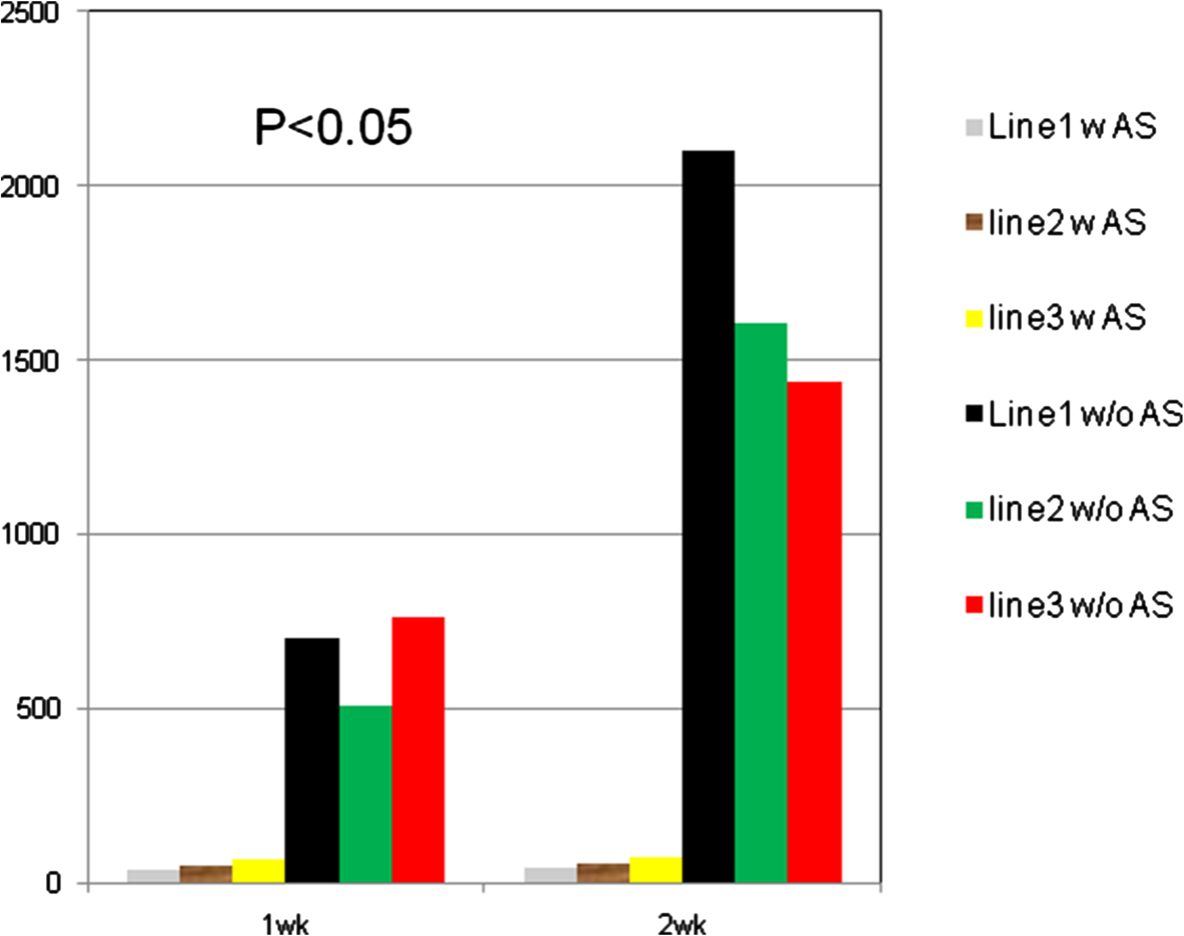
**Number of colonies formed by human ovarian cancer cells in soft agar treated with or without AS.** Human ovarian cancer cells(line1-3) after AS treatment in 15% serum-rich soft agar produced over 10-fold fewer colonies as compared to the cancer cells without AS treatment (P < 0.05).

As described in Methods, each clone was seeded in soft agar to check the inhibition of anchorage-independent cell growth by IGF-1R AS. Table 2 and Figure 5 showed the inhibitory effect of IGF-1R AS on the anchorage-independent growth of cancer cells. After AS treatment in 15% serum-rich soft agar, cancer cells produced over 10-fold fewer colonies compared to cancer cells without AS treatment (P < 0.05).

**Table 2:** Number of clonies formed in soft sugar

**Time**	**Cancer cells w/o AS**			**Cancer cells w AS**		
	**Line 1**	**Line 2**	**Line 3**	**Line 1**	**Line 2**	**Line 3**
1 wk	698	509	759	38	48	68
2 wk	2102	1608	1440	42	57	72

## Discussion

There are two main causes for high mortality rate of advanced ovarian cancer. The first is its tendency to spread into the abdominal cavity during its early stages. This intra-abdominal dissemination often makes complete resection of the disease quite difficult. The second is its acquired resistance to platinum-based drugs during cyclic chemotherapy [4,18]. Therefore, it has become essential to understand the molecular mechanism of ovarian cancer cell proliferation and growth and introduce new therapeutic modalities that may benefit these patients. The present study focuses on the role of IGF-1R in human epithelial ovarian cancer and the antitumor effect of IGF-1R AS. The pro-apoptotic function of IGF-1R might serve as a chemosensitizer, and therefore offers new insight and opportunity for the development of novel therapeutic strategies for the treatment of EOC.

In recent years, IGF-1R and its ligand IGF-1 have been considered not only to be growth factors but also potent survival factors [14,19,30,31]. IGF-1 is a progression factor able to bind the IGF-1R with high specificity. The activation of this receptor induces a cascade of intracellular tyrosine phosphorylations that culminate in the activation of transcription factors involved in the synthesis of proliferation-inducing proteins [14,30-32]. Under normal physiological conditions, the expression and activation of IGF-IR is tightly controlled. In this study using primary tumor tissue and cancer cells from patients, we showed striking over-expression of IGF-IR and IGF-1 in comparison to normal ovarian tissue samples. IGF-1 and IGF-1R both have been shown to be upregulated in multiple cancer cell types, affecting proliferation, differentiation, and metastasis. Primary cancer cells from patients with advanced epithelial ovarian cancer produced endogenous IGF-1, grew autonomously and proliferated in SFM. Their growth in SFM was further stimulated by the addition of IGF-1. These data confirm the existence of a functional autocrine loop in EOC. Treatment with IGF-1R AS markedly inhibited the proliferation of these cells both in SFM and in the presence of IGF-1. IGF-1R AS also inhibited anchorage-independent cell growth (Table 2, Figure 5). AS-treated ovarian cancer cells exhibited a corresponding reduction in the amount of detectable phosphorylated IGF-1R. Taken together, these data indicate that the IGF-1/IGF-1R system has a prominent role in the proliferation of human epithelial ovarian cancer cells.

Recent reports have implicated IGF-1R in programmed cell death [33,34]. The biochemical and molecular aspects of apoptosis have recently been delineated [35,36]. Resnicoff et al. have shown that an activated and overexpressed IGF-1R has a protective role in apoptosis [34], and that this function is independent of its mitogenic action [37]. There is evidence that cells may escape death either by an IGF-1R-mediated increase in Bcl-2 and Bcl-XL, or by a failure of p53 inhibition of mitogenic signaling by IGF-1R via secretion of IGF-binding proteins [38,39]. Another possibility is that the activation of IGF-1R modulates changes in the expression of death-inducing signaling complex proteins and/or receptors (MORT/FADD, MACH/FLICE, FAS, FAP-1,etc.) [8,40,41]. Interestingly, upon binding of IGF-1 to the IGF-1 receptor, a signaling cascade is put in motion resulting in increased gene activation, cell cycle progression, cell growth, differentiation and an anti-apoptotic effect. These events follow two distinct signaling pathways, with crosstalk and negative feedback loops creating a complex network of communication within the cell. One pathway is the PI3K/Akt pathway and the other is the Ras/MAPK pathway [42,43]. Both the PI3K-Akt-mTOR and Raf-MEK-MAPK pathways are thought to be important downstream signaling pathways ultimately leading to activation of mitogenic/anti-apoptotic transcription factors such as cFos and cJun [7,39]. Each signaling cascade takes a divergent path with some cross talk evident. In this study, inhibition of IGF-1R expression in primary ovarian cancer cells with an IGF-1R AS induced apoptosis. We tested the effect of the IGF-1R AS on EOC cells and observed a 35-40% rate of apoptosis. We found several of these IGF-1R signaling proteins had altered phosphorylation levels after antisense treatment in EOC. We measured these signaling proteins phosphorylation by western blot and observed variable inhibition of phosphorylated IRS-1, AKT, and MAP kinase expression in epithelial ovarian cancer cells by AS. This suggests that the apoptotic action of IGF-1R AS may represent the end result of the modulation of IGF-1R-dependent signaling cascade by decreased expression of IGF-1R in EOC cells. Additional investigations studying the interaction between apoptotic and anti-apoptotic signals should be done to provide new insights into the pathobiology of EOC.

With respect to drug targets, chemoresistance can also be triggered by overexpression of receptor tyrosine kinases, including ERB B1-4, IGF-1R, VEGFR 1–3 and PDGF receptor family members [44-46]. For instance, ERBB2 (also called HER 2) activates the small G protein RAS, leading to downstream MAPK signaling and proliferation as well as PI3K/AKT pathway and cell survival [46]. We hypothesize that IGF-1 and IGF-1R are important in the control of chemosensitivity and that their dysregulation may confer cisplatin chemoresistance in EOC. We found that rate of apoptosis induced by cisplatin alone was about 40% by TUNNEL assay. Notably, inhibition of IGF-1R by AS dramatically increased the sensitivity of ovarian cancer cells to the chemotherapeutic agent cisplatin compared with either agent alone. We observed a significant increase in apoptosis (60%) induced by the combination of AS and cisplatin. Our results indicate that IGF-IR–dependent signaling through the PI3K pathway mediates cisplatin resistance in ovarian cancer cells. Furthermore, cisplatin-resistant SKOV-3 [47] and OVCAR-4 [44] cells express both IGF-I and the IGF-IR [48].

## Conclusions

Our results provide strong evidence for an essential role for the IGF-IR signaling pathway mediating cisplatin resistance in ovarian cancer. This study is the first study to use primary clinical tumor material to provide a novel comprehensive insight into the relation between the IGF-1R signaling pathway and EOC tumorigenesis. IGF-1R is overexpressed in EOC and, acting in an autocrine way, can influence tumor cell growth, proliferation and apoptosis. These preclinical studies also provide the framework for future clinical evaluation of IGF-1R AS to treat EOC, either as a monotherapy or in combination with cisplatin, and holds potential to advance the development of therapies designed to overcome cisplatin resistance. To realize the potential of our findings to benefit ovarian cancer patients, it will be necessary to validate these results in a clinical study. We envision that the results of this study will translate into the clinic and aid the development of novel therapies targeting the IGF-IR pathways in ovarian cancer.

## Abbreviations

AS: Antisense oligonucleotide; S: Sense oligonucleotide; IGF-IR: Insulin-like growth factor I receptor; EOC: Epithelial ovarian cancer; SFM: Serum-free media; ELISA: Enzyme-linked immunosorbent assay; IHC: Immunohistochemistry.

## Competing interests

The authors declare that they have no competing interests.

## Authors’ contributions

JT and XX designed and wrote the manuscript. JL, GZ, YT, WT, JH performed experiment of molecular biology and clinical data collection. JPY helped to draft the manuscript. All authors read and approved the final manuscript.

## References

[B1] Pliarchopoulou K Pectasides D Epithelial ovarian cancer: focus on targeted therapy Crit Rev Oncol Hematol 2011 79 17 23 10.1016/j.critrevonc.2010.07.004 20674385

[B2] Markman M Sehouli J Levenback CF Chi DS Epithelial ovarian cancer: focus on targeted therapy J Oncol 2010 2010 171425 21804823 10.1155/2010/171425 PMC3143433

[B3] Malkasian GD Jr Melton LJ 3rd O'Brien PC Greene MH Prognostic significance of histologic classification and grading of epithelial malignancies of the ovary Am J Obstet Gynecol 1984 149 274 284 6328995 10.1016/0002-9378(84)90227-8

[B4] Ozols RF Bookman MA Connolly DC Focus on epithelial ovarian cancer Cancer Cell 2004 5 19 24 10.1016/S1535-6108(04)00002-9 14749123

[B5] Yee D Morales FR Hamilton TC Von Hoff DD Expression of insulin-like growth factor I, its binding proteins, and its receptor in ovarian cancer Cancer Res 1991 51 5107 5112 1717138

[B6] Ouban A Muraca P Yeatman T Coppola D Expression and distribution of insulin-like growth factor-1 receptor in human carcinomas Hum Pathol 2003 34 803 808 10.1016/S0046-8177(03)00291-0 14506643

[B7] LeRoith D Roberts CT Jr The insulin-like growth factor system and cancer Cancer Lett 2003 195 127 137 10.1016/S0304-3835(03)00159-9 12767520

[B8] Jin Q Esteva FJ Cross-talk between the ErbB/HER family and the type I insulin-like growth factor receptor signaling pathway in breast cancer J Mammary Gland Biol Neoplasia 2008 13 485 498 10.1007/s10911-008-9107-3 19034632

[B9] Sachdev D Yee D Disrupting insulin-like growth factor signaling as a potential cancer therapy Mol Cancer Ther 2007 6 1 12 17237261 10.1158/1535-7163.MCT-06-0080

[B10] Baxter RC Insulin-like growth factor (IGF)-binding proteins: interactions with IGFs and intrinsic bioactivities Am J Physiol Endocrinol Metab 2000 278 E967 E976 10826997 10.1152/ajpendo.2000.278.6.E967

[B11] Sayer RA Lancaster JM Pittman J Gray J Whitaker R Marks JR Berchuck A High insulin-like growth factor-2 (IGF-2) gene expression is an independent predictor of poor survival for patients with advanced stage serous epithelial ovarian cancer Gynecol Oncol 2005 96 355 361 10.1016/j.ygyno.2004.10.012 15661221

[B12] Belfiore A Frasca F IGF and insulin receptor signaling in breast cancer J Mammary Gland Biol Neoplasia 2008 13 381 406 10.1007/s10911-008-9099-z 19016312

[B13] Weiss JM Huang WY Rinaldi S Fears TR Chatterjee N Chia D Crawford ED Kaaks R Hayes RB IGF-1 and IGFBP-3: Risk of prostate cancer among men in the Prostate, Lung, Colorectal and Ovarian Cancer Screening Trial Int J Cancer 2007 121 2267 2273 10.1002/ijc.22921 17597108

[B14] Kurmasheva RT Houghton PJ IGF-I mediated survival pathways in normal and malignant cells Biochim Biophys Acta 2006 1766 1 22 16844299 10.1016/j.bbcan.2006.05.003

[B15] Moschos SJ Mantzoros CS The role of the IGF system in cancer: from basic to clinical studies and clinical applications Oncology 2002 63 317 332 10.1159/000066230 12417786

[B16] Slomiany MG Black LA Kibbey MM Tingler MA Day TA Rosenzweig SA Insulin-like growth factor-1 receptor and ligand targeting in head and neck squamous cell carcinoma Cancer Lett 2007 248 269 279 10.1016/j.canlet.2006.08.004 16996205

[B17] Resnicoff M Ambrose D Coppola D Rubin R Insulin-like growth factor-1 and its receptor mediate the autocrine proliferation of human ovarian carcinoma cell lines Lab Invest 1993 69 756 760 8264238

[B18] Berchuck A Kohler MF Boente MP Rodriguez GC Whitaker RS Bast RC Jr Growth regulation and transformation of ovarian epithelium Cancer 1993 71 545 551 8420675 10.1002/cncr.2820710209

[B19] Brokaw J Katsaros D Wiley A Lu L Su D Sochirca O de la Longrais IA Mayne S Risch H Yu H IGF-I in epithelial ovarian cancer and its role in disease progression Growth Factors 2007 25 346 354 10.1080/08977190701838402 18236213

[B20] Chien JR Aletti G Bell DA Keeney GL Shridhar V Hartmann LC Molecular pathogenesis and therapeutic targets in epithelial ovarian cancer J Cell Biochem 2007 102 1117 1129 10.1002/jcb.21552 17879946

[B21] Spentzos D Cannistra SA Grall F Levine DA Pillay K Libermann TA Mantzoros CS IGF axis gene expression patterns are prognostic of survival in epithelial ovarian cancer Endocr Relat Cancer 2007 14 781 790 10.1677/ERC-06-0073 17914107

[B22] Pollak M Targeting insulin and insulin-like growth factor signalling in oncology Curr Opin Pharmacol 2008 8 384 392 10.1016/j.coph.2008.07.004 18674638

[B23] Maloney EK McLaughlin JL Dagdigian NE Garrett LM Connors KM Zhou XM Blättler WA Chittenden T Singh R An anti-insulin-like growth factor I receptor antibody that is a potent inhibitor of cancer cell proliferation Cancer Res 2003 63 5073 5083 12941837

[B24] Dong J Demarest SJ Sereno A Tamraz S Langley E Doern A Snipas T Perron K Joseph I Glaser SM Ho SN Reff ME Hariharan K Combination of two insulin-like growth factor-I receptor inhibitory antibodies targeting distinct epitopes leads to an enhanced antitumor response Mol Cancer Ther 2010 9 2593 2604 10.1158/1535-7163.MCT-09-1018 20716637

[B25] Hadad SM Fleming S Thompson AM Targeting AMPK: a new therapeutic opportunity in breast cancer Crit Rev Oncol Hematol 2008 67 1 7 10.1016/j.critrevonc.2008.01.007 18343152

[B26] Fruman DA Edinger AL Cancer therapy: staying current with AMPK Biochem J 2008 412 e3 e5 10.1042/BJ20080823 18466113

[B27] Resnicoff M Coppola D Sell C Rubin R Ferrone S Baserga R Growth inhibition of human melanoma cells in nude mice by antisense strategies to the type 1 insulin-like growth factor receptor Cancer Res 1994 54 4848 4850 8069850

[B28] Hartmann W Kuchler J Koch A Koch A Friedrichs N Waha A Endl E Czerwitzki J Metzger D Steiner S Wurst P Leuschner I von Schweinitz D Buettner R Pietsch T Activation of phosphatidylinositol-3'-kinase/AKT signaling is essential in hepatoblastoma survival Clin Cancer Res 2009 15 4538 4545 10.1158/1078-0432.CCR-08-2878 19584164

[B29] Scherle PA Jones EA Favata MF Daulerio AJ Covington MB Nurnberg SA Magolda RL Trzaskos JM Inhibition of MAP kinase kinase prevents cytokine and prostaglandin E2 production in lipopolysaccharide-stimulated monocytes J Immunol 1998 161 5681 5686 9820549

[B30] O'Connor R Kauffmann-Zeh A Liu Y Lehar S Evan GI Baserga R Blättler WA Identification of domains of the insulin-like growth factor I receptor that are required for protection from apoptosis Mol Cell Biol 1997 17 427 435 8972223 10.1128/mcb.17.1.427 PMC231767

[B31] Pollak M Insulin and insulin-like growth factor signalling in neoplasia Nat Rev Cancer 2008 8 915 928 10.1038/nrc2536 19029956

[B32] Myers MG Jr Sun XJ Cheatham B Jachna BR Glasheen EM Backer JM White MF IRS-1 is a common element in insulin and insulin-like growth factor-I signaling to the phosphatidylinositol 3'-kinase Endocrinology 1993 132 1421 1430 10.1210/en.132.4.1421 8384986

[B33] LeRoith D Werner H Beitner-Johnson D Roberts CT Jr Molecular and cellular aspects of the insulin-like growth factor I receptor Endocr Rev 1995 16 143 163 7540132 10.1210/edrv-16-2-143

[B34] Resnicoff M Abraham D Yutanawiboonchai W Rotman HL Kajstura J Rubin R Zoltick P Baserga R The insulin-like growth factor I receptor protects tumor cells from apoptosis in vivo Cancer Res 1995 55 2463 2469 7758000

[B35] del PL Gonzalez-Garcia M Page C Herrera R Nunez G Interleukin-3-induced phosphorylation of BAD through the protein kinase Akt Science 1997 278 687 689 10.1126/science.278.5338.687 9381178

[B36] Virdee K Parone PA Tolkovsky AM Phosphorylation of the pro-apoptotic protein BAD on serine 155, a novel site, contributes to cell survival Curr Biol 2000 10 R883 10.1016/S0960-9822(00)00843-5 11114542

[B37] Harrington EA Bennett MR Fanidi A Evan GI c-Myc-induced apoptosis in fibroblasts is inhibited by specific cytokines EMBO J 1994 13 3286 3295 8045259 10.1002/j.1460-2075.1994.tb06630.x PMC395225

[B38] Virdee K Parone PA Tolkovsky AM Phosphorylation of the pro-apoptotic protein BAD on serine 155, a novel site, contributes to cell survival Curr Biol 2000 10 1151 1154 10.1016/S0960-9822(00)00702-8 10996800

[B39] Kalli KR Conover CA The insulin-like growth factor/insulin system in epithelial ovarian cancer Front Biosci 2003 8 d714 d722 10.2741/1034 12700030

[B40] Murata H Hresko RC Mueckler M Reconstitution of phosphoinositide 3-kinase-dependent insulin signaling in a cell-free system J Biol Chem 2003 278 21607 21614 10.1074/jbc.M302934200 12682058

[B41] Peruzzi F Prisco M Dews M Salomoni P Grassilli E Romano G Calabretta B Baserga R Multiple signaling pathways of the insulin-like growth factor 1 receptor in protection from apoptosis Mol Cell Biol 1999 19 7203 7215 10490655 10.1128/mcb.19.10.7203 PMC84713

[B42] Kanehisa M Goto S Hattori M Aoki-Kinoshita KF Itoh M Kawashima S Katayama T Araki M Hirakawa M From genomics to chemical genomics: new developments in KEGG Nucleic Acids Res 2006 34 D354 D357 10.1093/nar/gkj102 16381885 PMC1347464

[B43] Kanehisa M Goto S Furumichi M Tanabe M Hirakawa M KEGG for representation and analysis of molecular networks involving diseases and drugs Nucleic Acids Res 2010 38 D355 D360 10.1093/nar/gkp896 19880382 PMC2808910

[B44] Selvakumaran M Pisarcik DA Bao R Yeung AT Hamilton TC Enhanced cisplatin cytotoxicity by disturbing the nucleotide excision repair pathway in ovarian cancer cell lines Cancer Res 2003 63 1311 1316 12649192

[B45] Stewart DJ Mechanisms of resistance to cisplatin and carboplatin Crit Rev Oncol Hematol 2007 63 12 31 10.1016/j.critrevonc.2007.02.001 17336087

[B46] Eckstein N Platinum resistance in breast and ovarian cancer cell lines J Exp Clin Cancer Res 2011 30 91 10.1186/1756-9966-30-91 21967738 PMC3197542

[B47] Petru E Sevin BU Perras J Boike G Ramos R Nguyen H Averette HE Comparative chemosensitivity profiles in four human ovarian carcinoma cell lines measuring ATP bioluminescence Gynecol Oncol 1990 38 155 160 10.1016/0090-8258(90)90032-G 2387529

[B48] Burke F Relf M Negus R Balkwill F A cytokine profile of normal and malignant ovary Cytokine 1996 8 578 585 10.1006/cyto.1996.0077 8891439

